# Novel insights into the pathobiology of pulmonary hypertension in heart failure with preserved ejection fraction

**DOI:** 10.1152/ajpheart.00068.2024

**Published:** 2024-04-19

**Authors:** Vaishnavi Aradhyula, Rohit Vyas, Prabhatchandra Dube, Steven T. Haller, Rajesh Gupta, Krishna Rao Maddipati, David J. Kennedy, Samer J. Khouri

**Affiliations:** ^1^Department of Medicine, University of Toledo College of Medicine and Life Sciences, Toledo, Ohio, United States; ^2^Department of Pathology, Lipidomics Core Facility, Wayne State University, Detroit, Michigan, United States

**Keywords:** diastolic HF, HFpEF, PH, PH-HFpEF, PUFA

## Abstract

Heart failure (HF) with preserved ejection fraction (HFpEF) is the most common cause of pulmonary hypertension (PH) worldwide and is strongly associated with adverse clinical outcomes. The American Heart Association recently highlighted a call to action regarding the distinct lack of evidence-based treatments for PH due to poorly understood pathophysiology of PH attributable to HFpEF (PH-HFpEF). Prior studies have described cardiophysiological mechanisms to explain the development of isolated postcapillary PH (ipc-PH); however, the consequent increase in pulmonary vascular (PV) resistance (PVR) may lead to the less understood and more fatal combined pre- and postcapillary PH (cpc-PH). Metabolic disease and inflammatory dysregulation have been suggested to predispose PH, yet the molecular mechanisms are unknown. Although PH-HFpEF has been studied to partly share vasoactive neurohormonal mediators with primary pulmonary arterial hypertension (PAH), clinical trials that have targeted these pathways have been unsuccessful. The increased mortality of patients with PH-HFpEF necessitates further study into viable mechanistic targets involved in disease progression. We aim to summarize the current pathophysiological and clinical understanding of PH-HFpEF, highlight the role of known molecular mechanisms in the progression of PV disease, and introduce a novel concept that lipid metabolism may be attenuating and propagating PH-HFpEF.

## INTRODUCTION

Pulmonary hypertension (PH) is a progressive, debilitating disease with poor patient outcomes. One of the leading causes of PH is heart failure (HF) with preserved ejection fraction (HFpEF), or PH-HFpEF. As summarized in Table 1, PH-HFpEF is classified as World Health Organization (WHO) Group II PH because of left heart disease (PH-LHD) and carries a significant healthcare burden because of its high morbidity and mortality. Despite advances in our understanding of pathophysiology, the underlying molecular mechanisms are largely unknown. PH-HFpEF presents as a spectrum, initially presenting as isolated postcapillary PH (ipc-PH) that can progress to the severe combined pre- and postcapillary PH (cpc-PH). Cpc-PH consists of more extensive pulmonary vascular (PV) remodeling than ipc-PH and can trigger right ventricle (RV) dysfunction. Biological factors such as oxidative stress, inflammation, and metabolic derangements have been suggested to be associated with RV dysfunction in PH-HFpEF; however, the mechanisms underlying this association remain unclear. A significant unresolved question is why a subset of patients with HFpEF develop only ipc-PH, cpc-PH, or do not develop PH at all.

**Table 1.** Definitions of pulmonary hypertension by the World Health Organization

Group				
PAH; WHO Group I	PH-LHD; WHO Group II	PH due to chronic lung disease and/or hypoxia;WHO Group III	CTEPH; WHO Group IV	PH secondary to other diseases; WHO Group V
Examples				
Idiopathic PAH, heritable PAH, drug and toxin-induced, veno-occlusive disease, secondary to portal hypertension, congenital heart diseases, human immunodeficiency virus infection, connective tissue disease	Left ventricular systolic dysfunction, left ventricular diastolic dysfunction, valvular disease, congenital cardiac anomalies/outflow tract obstruction	Chronic obstructive pulmonary disease, interstitial lung disease, obstructive sleep apnea, alveolar hypoventilation disorders	CTEPH	Chronic hemolytic anemia, splenectomy, sarcoidosis, metabolic disorders
Subtypes				
Idiopathic/heritable	Isolated postcapillary PH/combined pre- and postcapillary PH	Nonsevere PH/severe PH	N/A	Hematologic/systemic disorders
Pathophysiology				
• Increased vasoconstrictors, decreased vasodilators • Extracellular matrix remod eling, vascular proliferation, and inflammatory cell infil tration • Right ventricular dysfunction	• Elevated left heart pres sure transmitted to pulmo nary vasculature leading to pulmonary vascular remodeling • Molecular mechanisms behind pulmonary vascu lar remodeling are largely unknown • Right ventricular dysfunction	• Chronic hypoxia leads to pulmonary vasoconstric tion, which leads to increased pulmonary vas cular resistance and PH • Inflammation and fibrosis contribute to remodeling • Right ventricular dysfunction	• Persistent clotting • Pulmonary vascular remodeling • Right ventricular dysfunction	• Variable pathophysiology

Pulmonary hypertension (PH) refers to any cause of high blood pressure in the lungs. The World Health Organization (WHO) defines five major groups of PH based on different etiologies. CTEPH, chronic thromboembolic pulmonary hypertension; N/A, not applicable; PAH, pulmonary arterial hypertension; PH-LHD, PH due to left heart disease.

Despite mechanistic similarities between PH-HFpEF and WHO Group I pulmonary arterial hypertension (PAH), such as elevated endothelin 1 (ET-1), lack of nitric oxide (NO), and decreased prostacyclin (PGI_2_), previous clinical trials that therapeutically targeted these pathways were beneficial in PAH, but demonstrated minimal or no effects in patients with PH-LHD (1). PH-LHD also consists of PH attributable to HF with reduced ejection fraction (HFrEF), or PH-HFrEF; however, PH-HFrEF differs from PH-HFpEF in etiology, cardiac remodeling, pathophysiology, comorbid diseases, and therapeutic response (1). PH-HFpEF is a lethal manifestation that lacks evidence-based approaches in diagnosis, prognosis, pathophysiology, and treatment (2). According to the 2022 American Heart Association (AHA) Science Advisory’s Call to Action, lack of standardization over diagnostic protocols, inconsistent interpretation of results, lack of reliable noninvasive diagnostic procedures, and poor mechanistic understanding of cpc-PH pathophysiology contribute to this growing epidemic (2).

Given the central role of inflammation in cardiovascular disease, examining the regulatory mechanisms that promote or resolve inflammatory processes may identify innovative therapeutic targets for PH-HFpEF. Incorporating noncardiac lipid contributors in the current paradigm is necessary for the understanding of PH-HFpEF to further close critical knowledge gaps. We review the current clinical understanding of PH-HFpEF, examine animal models that elucidate its pathophysiology, and describe novel inflammatory biomarkers contributing to pathological pulmonary vasculature changes.

## EPIDEMIOLOGY OF PH-HFpEF

Approximately half of all patients with HF have HFpEF, yet management of this form of HF remains a diagnostic and therapeutic challenge. Patients with HF can be divided into those with HFrEF and HFpEF. Regardless of ejection fraction (EF) value, these patients share many similar features across the EF spectrum, including abnormal left ventricular (LV) filling dynamics, diastolic dysfunction, impaired exercise tolerance, hospitalization for decompensated HF, and most importantly, reduced survival (3). Studies have shown that compared with HFrEF, the incidence of HFpEF increases more rapidly with age and is closely associated with chronic metabolic diseases such as obesity, type 2 diabetes mellitus, hypertension, pulmonary disease, and liver disease (4). Obesity is a powerful and independent predictor of HFpEF development; however, interestingly, after HFpEF diagnosis, the presence of obesity is protective in what is known as the obesity paradox (5). This paradox is inversed when obesity is concomitantly present with type 2 diabetes (5). More than 50% of patients with HFpEF have five or more metabolic syndrome (metS) comorbidities, and the highest risk of hospitalization and death is in patients with comorbid metabolic conditions (3). Although observational studies have reported increased prevalence among women, after adjusting for age and other risk factors, the incidence of HFpEF was numerically but not statistically higher in women compared with men (6). The severity of HFpEF also increases more rapidly with aging, suggesting a close interplay with age-related inflammatory progression compared with HFrEF (7). In addition, randomized clinical trials highlighted by the American College of Cardiology (ACC)/AHA hypertension treatment guidelines have shown that the treatment for hypertension reduces HFpEF incidence by up to 40% over 2–8 years (8).

### Risk Factors for PH-HFpEF

PH-LHD is associated with a 60% 5-years mortality rate and 50% 6-mo hospitalization rate (9). PH is a strong predictor of morbidity and mortality in patients with HFpEF. Reports from the Olmsted County Heart Failure Surveillance Study have shed light on the prevalence of PH-HFpEF in both the general community and in patients with HFpEF (10). These studies showed that aging, increased LV pressures, and systemic vascular stiffening were associated with increasing pulmonary arterial systolic pressure (PASP) (10). PASP was the strongest predictor of mortality in comparison with established markers, and these outcomes were similarly reflected in the general population as well (10). Overall, the prevalence of PH-HFpEF has been shown to range from 36 to 83% (10). The wide range can be attributed to the variability of studies based on diagnostic methods, definitions, study design, and populations. PH-HFpEF is a poor prognostic indicator, with PASP greater than 44 mmHg on echocardiography being associated with increased 5-years mortality, independent of the severity of HF (11).

As shown in Table 2, Stolfo et al. (12) identified that female sex, obesity, and hypertension were more prevalent in patients with HFpEF compared with HFrEF. Fayyaz et al. (16) also described body mass index (BMI) and dyslipidemia as risk factors between HF-PH versus no HF-PH. In addition, Rezaee et al. (13) observed age, BMI, and type 2 diabetes as risk factors more significant in patients with ipc-PH versus cpc-PH. Opitz et al. (14) identified that compared with WHO Group I idiopathic PAH, patients with PH-HFpEF had older age, higher BMI, more frequent hypertension, and type 2 diabetes as significant risk factors. Finally, Thenappan et al. (15) established that female sex carries a higher risk burden for PH-HFpEF compared with HFpEF.

**Table 2.** Demographic risk factors of PH-HFpEF

	Stolfo et al. (12)		Rezaee et al. (13)		Opitz et al. (14)		Thenappan et al. (15)	
	HFpEF	HFrEF	Ipc-PH	Cpc-PH	PH-HFpEF	PAH	HFpEF	PH-HFpEF
*n*	18,165	40,893	457	54	226	421	45	100
Age, yr	80	76	66.3 ± 11.3	63.9 ± 10.5	73.2 ± 8.3‡	61.5 ± 17.3	67 ± 11	64 ± 13
Female, *n* (%)	9,731 (53.6)*	11,846 (29)	198 (43.3)	26 (48.2)	140 (61.9)	250 (59.4)	26 (58)§	82 (82)
BMI, kg/m^2^	27 [24, 31]	26 [23, 30]	32.0 [N/A]	34.3 [N/A]	29.6 [25.7, 34]‡	26 [23.3, 29.8]	DNP	DNP
Conditions, *n* (%)								
Obesity	3,259 (31.2)*	5,875 (23.8)	87 (23.8)†	14 (31.8)	106 (47.1)‡	99 (23.5)	22 (49)	46 (46)
Hypertension	12,313 (67.8)*	20,401 (49.9)	287 (64.6)	34 (64.2)	208 (91.9)‡	182 (43.2)	33 (77)	79 (79)
Type 2 diabetes	4,291 (27.1)	10,125 (24.8)	161 (36.6)	20 (37.0)	93 (41.2)‡	45 (10.7)	12 (28)	37 (37)

Values are means ± SD, *n*, number of patients (%), or medians [interquartile ranges]. Cpc-PH, combined pre- and postcapillary pulmonary hypertension; DNP, data not provided; HFpEF, heart failure with preserved ejection fraction; HFrEF, heart failure with reduced ejection fraction; ipc-PH, isolated postcapillary pulmonary hypertension; PAH, pulmonary arterial hypertension; PH-HFpEF, pulmonary hypertension in the setting of HFpEF; N/A, not applicable.

* *P* < 0.05 vs. HFrEF; †*P* < 0.05 vs. cpc-PH; ‡*P* < 0.001 vs. PAH; §*P* < 0.05 vs. PH-HFpEF.

### Cpc-PH and ipc-PH in HFpEF

Although ipc-PH is the most prevalent form of PH-HFpEF (10), cpc-PH is considered a more serious form than ipc-PH, with prevalence as high as 69% in patients with HFpEF compared with 47% in HFrEF (16). Studies have demonstrated mortality rates in HF with cpc-PH to be 2.5 times higher compared with ipc-PH and 5.3 times higher compared with no PH (13). Assad et al. (17) found that patients with cpc-PH were more likely younger, female, with more severe PV disease, higher brain-type natriuretic peptide (BNP) values, higher glycated hemoglobin (HbA1c) values, and worse renal function despite similar comorbidities and LV remodeling. Innovative solutions to understand PV remodeling via molecular profiling of transpulmonary blood samples are critical to detail the mechanisms behind cpc-PH due to the poor prognosis and poorly anticipated response to PAH therapy (17).

## HEMODYNAMIC DEFINITIONS OF PH-HFpEF

HFpEF is characterized by normal LV ejection fraction (LVEF), normal LV end-diastolic volume, and abnormal diastolic function. These findings often accompany LV concentric remodeling/hypertrophy and left atrial (LA) enlargement. The 2022 ACC/AHA/Heart Failure Society of America (HFSA) guidelines for the management of HF define HFpEF as an LVEF of ≥50% (18). A key hemodynamic feature that differentiates PH-HFpEF from WHO Group I PAH is PA wedge pressure (PAWP) elevation >15 mmHg.

**Figure 1.**
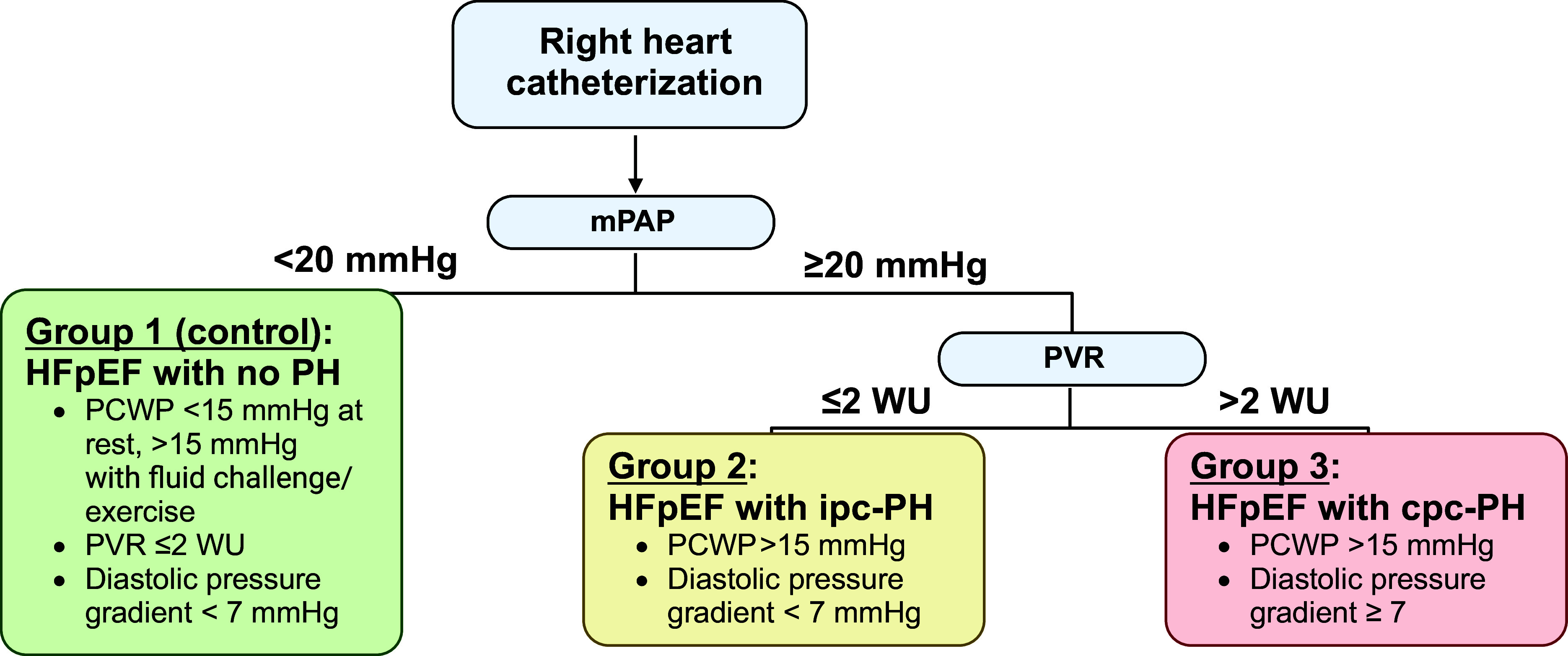
Hemodynamic definitions of pulmonary hypertension (PH) due to heart failure (HF) with preserved ejection fraction (HFpEF). PH due to HFpEF (PH-HFpEF) can be classified into compensated HFpEF, HFpEF with isolated postcapillary PH (ipc-PH), or HFpEF with pre- and postcapillary PH (cpc-PH). With the use of right heart catheterization, the gold standard tool for diagnosis, the mean pulmonary arterial pressure (mPAP) can be identified. According to the 2022 European Society of Cardiology/European Respiratory Society Guidelines for the Diagnosis and Treatment of Pulmonary Hypertension, if the mPAP is <20 mmHg, the patient is characterized as having compensated HFpEF, defined as a pulmonary capillary wedge pressure (PCWP) < 15 mmHg at rest or >15 mmHg on provocation with fluid challenge or exercise, pulmonary vascular resistance (PVR) ≤ 2 wood units (WU), and transpulmonary gradient (TPG) ≤ 12 mmHg. Conversely, if the mPAP is >20 mmHg, patients can be defined as either having HFpEF with ipc-PH or with cpc-PH. The defining factor between ipc-PH vs. cpc-PH is PVR. A PVR of ≤2 WU is quantified as ipc-PH, where patients have increased PAWP >15 mmHg, but normal or low TPG of ≤12 mmHg. A PVR >2 is defined as cpc-PH, characterized by PAWP >15 mmHg, but TPG >12, signifying a distinctive hemodynamic process from ipc-PH. Figure was created with a licensed version of BioRender.com.

Hemodynamic definitions of ipc-PH and cpc-PH overlap and are also distinctive. Identical demographic groups with ipc-PH and cpc-PH demonstrate higher mortality in cpc-PH when defined according to PV resistance (PVR) (17, 19). As shown in Fig. 1, according to the 2022 European Society of Cardiology/European Respiratory Society Guidelines for the Diagnosis and Treatment of Pulmonary Hypertension, the current hemodynamic definition of ipc-PH is mean pulmonary arterial pressure (mPAP) >20 mmHg, PAWP >15 mmHg, and PVR ≤2 wood units (WU) (19). The current definition of cpc-PH is mPAP >20 mmHg, PAWP >15 mmHg, and PVR >2 WU, making PVR as the discerning criteria between ipc-PH versus cpc-PH (19).

### Right Ventricular-Pulmonary Artery Coupling

Right ventricular (RV)-pulmonary artery (PA) coupling is a well-established tool that characterizes the interaction between myocardial contractile function and the load opposed by arterial circulation. RV-PA coupling can be evaluated by the ratio of the tricuspid annular plane systolic excursion (TAPSE) to the PASP. This ratio of TAPSE:PASP has been found to be worse in cpc-PH compared with ipc-PH (20). More importantly, TAPSE:PASP has been recently implicated as a powerful prognostic factor in HF and valvular heart disease (21, 22). This parameter is investigational and has not been incorporated into any proposed diagnostic criteria for cpc-PH. Echocardiographic assessment of PVR is possible but is dependent on obtaining an adequate imaging window and a measurable tricuspid regurgitation jet. Given the crescent shape of the RV, three-dimensional echocardiography and cardiac magnetic resonance imaging have also been proposed to assess RV in patients with PH-HFpEF (23).

### Pulmonary Arterial Compliance

Finally, LA hypertension and PVR can impact both PA compliance and elastance, defined by PASP-to-stroke volume ratio. These factors better represent the total RV afterload compared with precapillary parameters and are better predictors of PH-HFpEF; however, these factors are not useful to distinguish between ipc-PH and cpc-PH. PVR increases over time with chronic elevation of the intracardiac pressure, which widens the pressure difference between the LA and the PA, or the transpulmonary gradient (TPG). TPG is normally <12 mmHg. As the PVR and TPG increase, RV afterload also increases gradually and ultimately leads to RV dysfunction and failure.

## DIAGNOSIS OF PH-HFpEF

The current diagnosis of PH-HFpEF requires comprehensive clinical, echocardiographic, and hemodynamic assessments, as it can be easily misdiagnosed for WHO Group I PAH. Supportive laboratory evaluation typically shows elevated BNP levels. Transthoracic echocardiography (TTE) is the first step in the evaluation of patients with suspected PH to differentiate between HFpEF and HFrEF (24). Doppler echocardiography may show elevated PASP with variable accuracy between studies due to issues with image quality, limited acoustic windows, or lack of measurable tricuspid regurgitation (25). Pulmonary function testing and ventilation perfusion scanning can be useful to differentiate the subtypes of PH, such as WHO Groups III and IV PH (26).

Via the Bernoulli equation, TTE can estimate PASP by measuring peak tricuspid regurgitation velocity using estimation of right atrial (RA) pressures via inferior vena cava size; however, PASP estimates can have reduced accuracy due to inability to obtain adequate images, lack of measurable tricuspid regurgitation velocity, or inability to visualize the inferior vena cava (27). Therefore, right heart catheterization (RHC) is essential and is the gold standard for PH-HFpEF diagnosis and to differentiate between ipc-PH and cpc-PH. RHC provides direct hemodynamic data that can determine cardiac output, evaluate intracardiac shunts and valvular dysfunction, provide direct intracardiac chamber pressure measurements, and correctly classify PH-LHD versus other causes of PH (26, 28).

PAWP, which is the core value to diagnose PH-HFpEF, is elevated in PH-HFpEF and typically low or normal in WHO Group I PAH. PAWP is usually measured at end expiration during normal respiration; indeed, presence of a characteristic “wedge” tracing and a sample oxyhemoglobin saturation greater than 90% can confirm a true wedge position during RHC. However, many of these patients are already on diuretic therapy, which may result in a normal PAWP. In such cases, provocative maneuvers can be performed during RHC (28, 29). Such maneuvers can include fluid challenge or exercise during RHC. Recent investigations have demonstrated the utility of passive leg raise and exercise for the diagnosis of HFpEF among subjects with normal resting PAWP (30). Robbins et al. (29) confirmed that more than 20% of patients initially diagnosed with PAH by RHC were reclassified to PH-LHD with elevated PAWP after receiving 500 mL rapid fluid challenge.

A different pulmonary hemodynamic response is seen in PH-HFpEF versus PH-HFrEF for a similar average PAWP, suggesting divergent PV pathophysiological changes (31). These changes can be further defined with combining exercise-stress echocardiography with exercise testing, which evaluate the role of LA function and RV-PA uncoupling in prognosis across the HF spectrum (32). Magnetic resonance imaging-derived indices correlate with RHC results and exhibit differences in function, motion, and deformation of LV and RV between PH-HFpEF and HFrEF (33).

## PATHOPHYSIOLOGY OF PH-HFpEF

**Figure 2.**
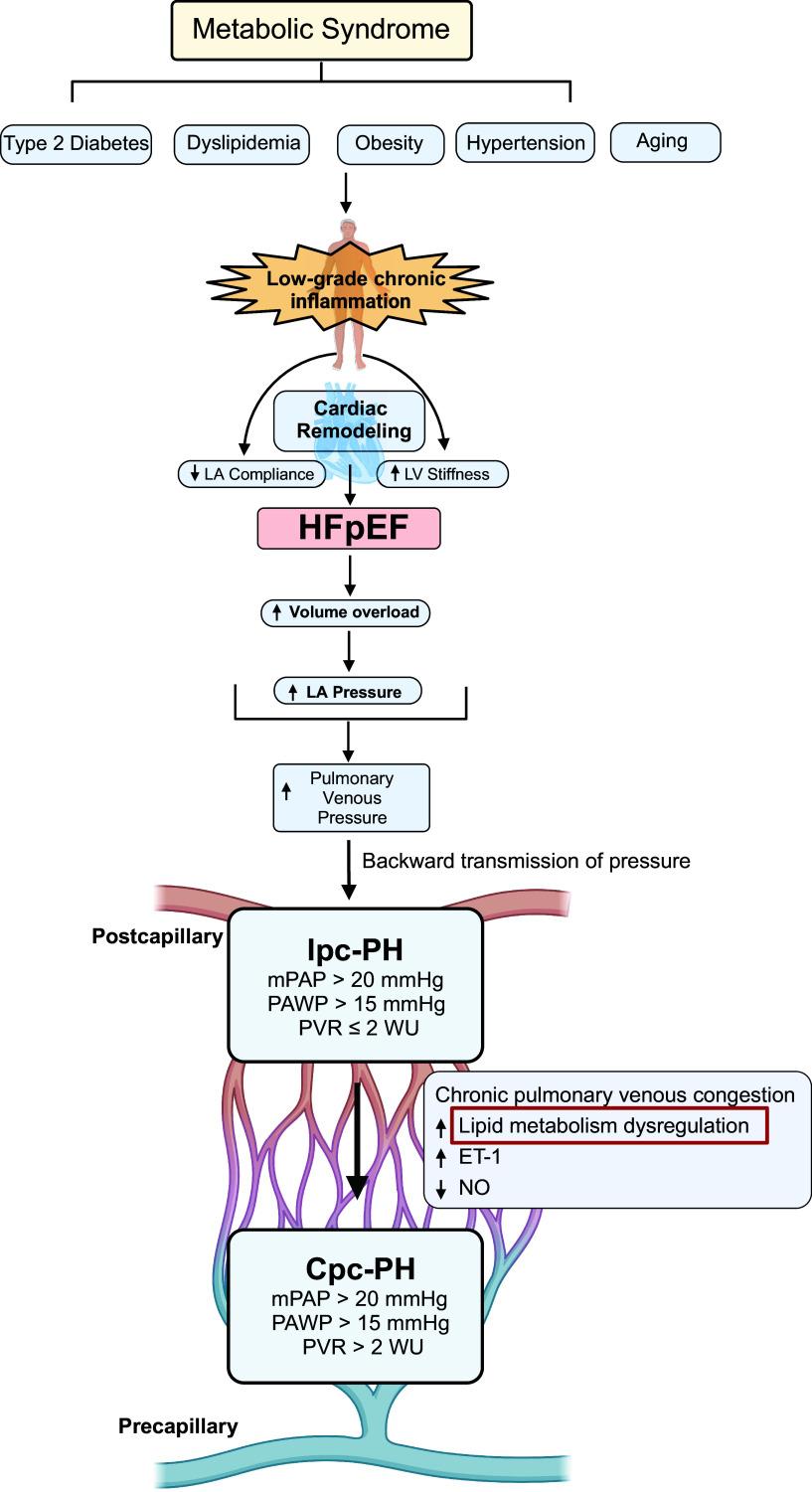
Development of pulmonary hypertension (PH) in heart failure (HF) with preserved ejection fraction (HFpEF). Pathophysiology of PH in HFpEF (PH-HFpEF) is complex. Features of metabolic syndrome (metS), such as type 2 diabetes, dyslipidemia, obesity, aging, and hypertension have been well studied to be risk factors and comorbidities of HFpEF via cardiac remodeling and diastolic dysfunction. Subsequent volume overload and increase in left atrial (LA) pressures lead to increased pulmonary venous pressures and isolated postcapillary PH (ipc-PH). Transition from ipc-PH to the more severe, irreversible pulmonary vascular (PV) remodeling and increased PV resistance (PVR) seen in combined pre- and postcapillary PH (cpc-PH) is not well studied. We predict that low-grade inflammation, through lipid metabolism dysregulation, decreased nitric oxide (NO) bioavailability, and increased endothelin-1 (ET-1) contribute to cpc-PH in HFpEF. LV, left ventricular; mPAP, mean pulmonary artery pressure; PAWP, pulmonary artery wedge pressure; WU, wood units. Figure was created with a licensed version of BioRender.com.

The pathophysiology of PH-HFpEF (Fig. 2) is complex, multifactorial, and multisystemic. In patients with HFpEF, the heart is unable to fill properly during diastole because of hypertension-induced ventricular hypertrophy and inflammation-induced myocardial fibrosis. Cardiac output, however, remains preserved or near normal because of compensation by abnormal myocardial active relaxation and increased LV stiffness, elevating LV and LA pressures. The impaired cardiac relaxation in HFpEF is attributed to multiple causes. Molecular changes in key calcium handling proteins in the LV such as sarcoplasmic reticulum calcium ATPases can lead to reduced calcium removal and cause LV diastolic dysfunction (34). In addition, increased ventricular load leads to a stiffened arterial system, resulting in ventricular-vascular uncoupling. LV wall stress leads to cardiomyocyte hypertrophy and interstitial fibrosis (2). In response to hypoxemia, cardiomyocytes release proinflammatory cytokines and chemokines, which initiate cardiac fibrosis and myocardial stiffness. Consequently, the pulmonary vasculature is exposed to a pressure challenge and acute pulmonary edema. Sustained backward pressure transmission increases RV load, triggers PH, and leads to classic symptom exacerbation. Although the primary anomaly is abnormal diastology and subtle irregularities in systolic function, limitations in LV systolic reserve, LA size and compliance, pulmonary vasculature, RV function, arterial stiffening, peripheral impairments, and extensive molecular and inflammatory changes contribute to HFpEF. In contrast, the HFrEF phenotype presents LA enlargement and increased LA pressures due to severe mitral regurgitation, highlighting that varying comorbidities present convergent PV changes. In fact, atrial fibrillation-predominant HFpEF presents similarly to PH-HFrEF and is associated with mitral regurgitation, higher PVR, and worse outcomes (35).

### Biomechanics of ipc-PH and cpc-PH

The pulmonary vascular bed is initially quite distensible; however, the sensitivity of the PVR and PV compliance to the elevated left-sided pressures introduces new hemodynamic consequences of LHD. Tedford et al. (36) studied the relationship between RV afterload and pulmonary circulation in PH, and this study provided further insight into the compensatory role of increasing pulmonary compliance on reducing RV dysfunction. Specifically, this study demonstrated that PH and fibrosis do not change the relationship between PV resistance and compliance, which could explain why resistance-decreasing therapies do not reduce consequential RV afterload (36). In addition, increased PAWP augments complexity by increasing RV afterload further and may contribute to RV dysfunction. The role of PAWP can be attributed to PH-LHD and highlights key mechanistic differences between PAH and Group II PH.

PH-HFpEF can be broken into a continuum that starts with patients who are asymptomatic with normal intracardiac pressures at rest. As end diastolic pressure increases, PH develops and is divided into two components: the ipc-PH component and cpc-PH component (14). In ipc-PH, the mPAP is elevated solely from the passive transmission of increased left-sided filling pressures to the pulmonary circulation. The mPAP increases slowly, and capillary barotrauma does not occur, as occurs with pulmonary edema. Primary adaptive changes include increased parietal thickness, collagen deposition, and lumen narrowing of the pulmonary veins in a process known as “arteriolarization” (16). This process is reversible once the LA decompression is removed, if the arteriolarization has not yet occurred (16). In cpc-PH, the mPAP is elevated from passive transmission of chronic, severe LA hypertension and dysfunction leading to PV remodeling and increased PVR. Initially, pulmonary arterioles and small arteries are affected, but eventually medium and larger arteries are affected, which further compromises the compliance of the PA bed (37).

Recent theories suggest the role of vascular biomechanical forces, such as cyclic stretch and wall lumen shear stress, which are transduced into biological signals in a process called mechanotransduction (38). Mechanotransduction transmits and induces biological signals that drive vascular remodeling, including hypertrophy, hyperplasia, apoptosis, and extracellular matrix (ECM) synthesis and degradation (38). This remodeling decreases vessel compliance via cytoskeleton remodeling and increased intimal thickness, which has been shown in human PV tissues as a key histological feature of PH-LHD (16). The combination of intimal thickness and rise in mPAP with subsequent increase in cyclic stretch injures pulmonary capillaries and leads to stress failure. The chronic elevated pulmonary pressure and decreased venous mechanical properties eventually lead to further irreversible PA remodeling, inflammation-induced remodeling, upregulation of ET-1, and downregulation of vasodilatory NO, marking the transition to cpc-PH (14, 16). Interestingly, these characteristics of cpc-PH more closely resemble PAH than ipc-PH; however, pulmonary venules are more extensively involved in the vascular remodeling in cpc-PH than in PAH (37).

### Abnormal Calcium Handling

Although calcium regulation and defective excitation-contraction coupling underlie abnormal contractility in HFrEF, very little is known about how calcium handling is regulated in HFpEF. Levosimendan, a calcium-sensitizing cardiotonic agent, has been shown to benefit mouse models of HFpEF with comorbid metS by reversing mitochondrial function and decreasing reactive oxygen species (ROS) production (39). In fact, Levosimendan also reduced PAWP in a phase II clinical trial of patients with PH-HFpEF, suggesting a key role of calcium desensitization in the pathogenesis of PH-HFpEF (40). The drug was also shown to induce venodilation in the systemic and pulmonary vasculature, stimulating positive effects on RV function (40).

### RV Dysfunction in cpc-PH

Cpc-PH leads to reduced RV function as PVR increases. As the RV normally ejects into the low impendence and highly distensible PV bed, it has a higher afterload sensitivity compared with the LV. Despite adaptive compensatory hypertrophy and increased contractility, the RV eventually develops systolic dysfunction because of afterload mismatch. The consequences of RV failure include increased central venous pressure and systemic circulation congestion, which lead to a myriad of systemic disorders that increase morbidity and mortality. The precapillary component of cpc-PH introduces the increased afterload and remodeling that accelerates HFpEF (41). Multiple studies have investigated the prevalence and prognostic relevance of RV dysfunction in HFpEF and found RV dysfunction to be a powerful predictor of mortality in HFpEF (42).

### Plexiform Lesions Distinguishing cpc-PH and PAH

Assad et al. (17) demonstrated shared genetic variants characterizing WHO Group I PAH and Group II cpc-PH, suggesting shared vascular remodeling pathways; however, the cause of PVR is a key pathophysiologic factor that can distinguish PH-LHD from PAH. Plexiform lesions, characterized by typical smooth muscle proliferation and hypertrophy, highlight the core pathological findings and etiology of increased vascular resistance and remodeling in PAH. Conversely, canine models of pace-induced HF have shown true increase in membrane and capillary arterial wall thickness, characterized by changes in basement membrane composition and deposition of type IV collagen (43). These changes lead to increased PVR or cpc-PH rather than the plexiform complex seen in typical PAH (43). Patients with PH-HFpEF display multifactorial PV remodeling, correlating with intimal thickening in pulmonary veins. This intimal thickening is seen in greater than 50% of patients with HFpEF, indicating an unknown variability (16).

### Comorbid Lung Disease

Chronic obstructive pulmonary disease (COPD) and HF share many risk factors and co-occur, challenging diagnosis of PH-HFpEF versus comorbid COPD and HF. Patients with COPD are more commonly predisposed to HFpEF than HFrEF because of chronic hypoxia and PV remodeling, and misdiagnosis is common (44). Key differences lie between COPD and PH-HFpEF, such as RA enlargement, and higher mean RA pressure, a predictor of mortality in COPD (45); however, cpc-PH presents with similar hemodynamic features, leading to a perplexing clinical case. Due to the clinical implications and distinct therapeutic targets of these disease conditions, succinct identification of PH-HFpEF is important to develop evidence-based therapeutic guidelines.

## VASOACTIVE NEUROHORMONAL ENDOTHELIAL MEDIATORS

Endothelial dysfunction is thought to play a central role in the pathogenesis of PH by mediating structural changes in the pulmonary vasculature. Evidence suggests that endothelial cells participate in the process of vascular injury and immune activation by existing in a circulating form (46). Increased circulating cells have been demonstrated to predict adverse cardiovascular events in acute coronary syndromes and vascular injury (46). Endothelial cells induced to undergo premature senescence were observed to have higher RV systolic pressure and increased RV mass compared with controls, indicating exacerbated PH (47). The most credited hypothesis of HFpEF pathogenesis suggests that HFpEF and comorbidity burden impair the coronary endothelium, activating complex molecular pathways that converge to generate myocardial stiffening, fibrosis, and diastolic dysfunction (48). The lack of human HF pulmonary vascular tissue poses a significant shortcoming to PH-HFpEF biomolecular research in understanding PA endothelial response (2).

### Decreased Nitric Oxide Bioavailability

Studies have demonstrated that endothelial dysfunction in PH-HFpEF is caused by an imbalance between nitric oxide (NO) and ET-1 signaling, causing cardiac nitrosative and oxidative stress (49). A series of studies investigating the effects of NO blockade in pulmonary circulation have shown that endothelium-derived NO mediates basal PV tone and dilation to endothelium-dependent stimuli (50). In healthy humans, systemic infusion of acetylcholine, a NO synthase agonist, increases local pulmonary blood flow, whereas infusion of a NO synthase inhibitor decreases pulmonary blood flow (50). Decreased NO bioavailability is observed in patients with HFpEF, which may explain the impaired vasodilation response to acetylcholine stimulation in the coronary capillary bed (51). Endothelial-derived NO also inhibits smooth muscle proliferation and hypertrophy along with PGI_2_, preventing platelet aggregation and adhesion (52). Pressure-induced cyclic stretch, previously described as a contributor to wall stress, also participates by increasing endothelial NO synthase phosphorylation in human umbilical vein endothelial cells, which increases IL-6 and cytokine release (53). Despite decreased NO bioavailability being a longstanding paradigm in the pathogenesis of PH-HFpEF, the clinical utility of this mechanism necessitates further study. Several recent trials have called into question the link between NO bioavailability and the pathogenesis of PH-HFpEF (1), and recent trials of agents targeting deficient NO signaling did not improve symptoms or outcomes (54–56). Thus, the therapeutic role of NO necessitates further study.

### Increased Endothelin-1 and Mortality

In PH-HFpEF, LA hypertension causes pulmonary edema, which further activates endothelin-1 (ET-1) expression and decreases NO and BNP activity (57). Chowdhury et al. (58) investigated the potential role of ET-1 in the pathogenesis of PH-HFpEF and demonstrated that ET-1 is predictive of 1-year HF hospitalization in patients with HFpEF. Increasing ET-1 levels were also associated with long-term mortality and worsening PV remodeling in PH-HFpEF (58). Specifically, higher ET-1 from wedge samples (ET-1 produced in the pulmonary vasculature) was associated with PH, elevated PVR, and increased risk of hospitalization (58). Other studies have also demonstrated elevated ET-1 in patients with cpc-PH, implicating ET-1 as a potential contributor to and prognostic biomarker for the development of cpc-PH (59). Obokata et al. (57) also demonstrated elevated ET-1 and adrenomedullin, a counterregulatory neurohormone in patients with HFpEF; these data are consistent with the hypoxia-induced upregulation of the pulmonary endothelial mechanisms that contribute to the transition from ipc-PH to cpc-PH. Reactive oxygen species (ROS) production has also been shown to increase ET-1 release in human umbilical vein endothelial cells, further suggesting the role of endothelial cell factors influencing intimal thickness and remodeling (53). ET-1 may also be a potential therapeutic target, with studies demonstrating that dual inhibition of ET-1 receptors improved HFpEF by reducing adverse cardiac hypertrophy and PV remodeling (60). These data strongly suggest that ET-1 potentiates endothelial dysfunction and vasoconstriction via lipid peroxidation and oxidation; however, trials have not shown favorable outcomes (41). Unfortunately, PH-HFpEF treatment guidelines also strongly recommend against using PAH therapies outside of clinical trials (24).

## METABOLIC SYNDROME AND INFLAMMATION

The lack of human HF PV tissue poses a significant shortcoming to PH-HFpEF research. Without molecular mechanisms, it is difficult to profile diagnostic markers that may be causing inflammatory processes in PH-HFpEF. Decreased NO bioavailability and impaired NO-mediated relaxation has been directly linked to ROS from metS-induced systemic inflammation; indeed, this can impair contractility of cardiomyocytes and deteriorate diastolic function (61). Multiple studies have demonstrated how intramyocardial lipid accumulation in patients with metS contributes to diastolic dysfunction, which, acting in concert with HFpEF, progresses to PH-HFpEF (62). Lai et al. (63) induced PH-HFpEF in a metS model of double-leptin receptor defect; interestingly, PH-HFpEF in early stages was responsive to nitrite and metformin treatment, but PH-HFpEF in later stages, likely progressed to cpc-PH, was not treatable with nitrite and metformin. This suggests additional deleterious processes other than NO bioavailability contributing to cpc-PH.

**Figure 3.**
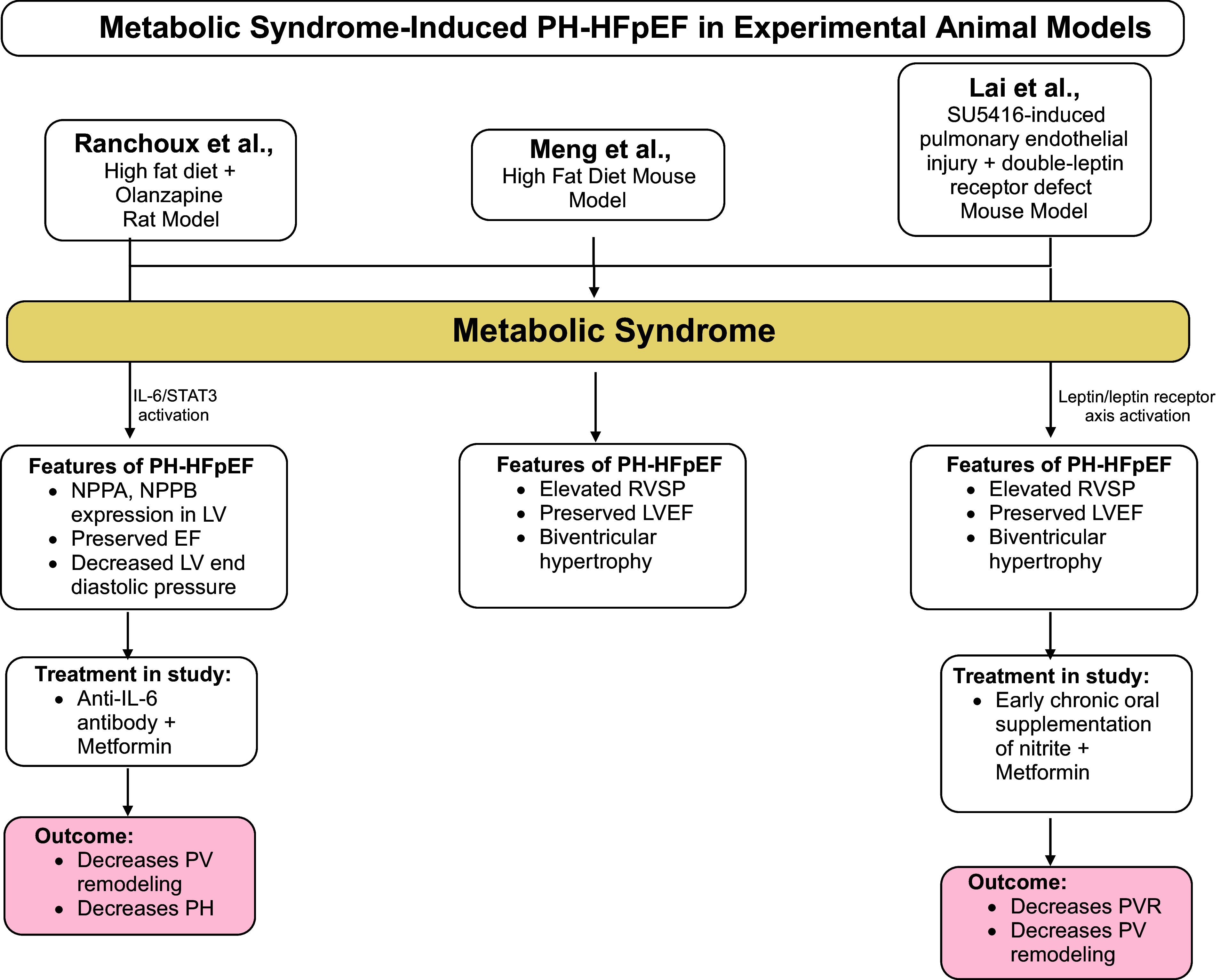
Metabolic syndrome-induced pulmonary hypertension (PH)-heart failure (HF) with preserved ejection fraction (PH-HFpEF) in experimental animal models. PH-HFpEF has been strongly associated with features of metabolic syndrome (metS), including hyperglycemia, adiposity, and insulin resistance. Many studies have explored the association of metS with PH-HFpEF, specifically via the use of animal models. Ranchoux et al. (64) developed a metS rat model through a high-fat diet and olanzapine-induced hyperglycemia and diastolic dysfunction via supracoronary aortic banding. This rat model developed features of PH-HFpEF. Anti-IL-6 antibodies and metformin treatment reversed features of PH, such as pulmonary vascular (PV) remodeling. Meng et al. (65) demonstrated how a metS model via a high-fat diet induced features of PH-HFpEF; no treatment modality was used in this study to reverse the adiposity. Lai et al. (63) studied how pulmonary endothelial injury in leptin receptor-deficient obese mouse model of metS induced PH-HFpEF features. Treatment with nitrite and metformin reversed the features of metS, including hyperglycemia and glucose intolerance, and increased adiponectin levels, a key protector of metabolic and vascular diseases. However, more severely affected PH-HFpEF rats, likely progressed to pre- and postcapillary PH (cpc-PH), did not benefit from nitrite or metformin treatment, suggesting the need for early mets therapeutic intervention as nitric oxide (NO) bioavailability may play a less predominant role in cpc-PH pathogenesis in HFpEF. EF, ejection fraction; LV, left ventricle; LVEF, left ventricle ejection fraction; RVSP, right ventricular systolic pressure; PVR, pulmonary vascular resistance. Figure was created with a licensed version of BioRender.com.

Here, we describe the utility of preliminary animal models of metS and PH-HFpEF that have been proposed to contribute to PH-HFpEF pathogenesis (Fig. 3). Elevated circulating inflammatory biomarkers such as C-reactive protein (CRP), interleukin-6 (IL-6), and tumor necrosis factor-α (TNF-α) are more pronounced in HFpEF than HFrEF, suggesting unique inflammatory signaling substantiating the comorbidity-inflammation paradigm in HFpEF (66). Several inflammatory biomarkers have been found to be better predictors of HFpEF severity and outcomes than natriuretic peptides, with endomyocardial biopsies from patients with HFpEF showing inflammatory cell infiltration correlating with diastolic dysfunction (67).

### Interleukin-6

Elevation of proinflammatory cytokine interleukin-6 (IL-6) has been shown to experimentally increase PA smooth muscle cell (PASMC) proliferation through STAT3 (signal transducer and activator of transcription 3) activation (68). This proinflammatory state also induces insulin resistance by increasing free fatty acid (FFA) concentration and interfering with the anti-inflammatory effect of insulin. Insulin has been studied to suppress NF-κB binding activity, ROS generation, and other proinflammatory transcription factors (69). Therefore, insulin resistance, as seen in type 2 diabetes, can induce a proinflammatory state and further increase IL-6. Ranchoux et al. (64) developed a PH-HFpEF rat model via olanzapine-induced hyperglycemia, high-fat diet-induced metS, and supracoronary aortic banding-induced diastolic dysfunction. Interestingly, only the rats with both metS and diastolic dysfunction displayed an increase in PVR and adverse PV remodeling, reflecting pathophysiological characteristics of PH-HFpEF (64). This combination of diastolic dysfunction and metS also induced a significant proinflammatory state via increased expression of IL-6. In fact, reducing IL-6 by anti-IL-6 antibodies and metformin reversed PV remodeling (64). This study strongly emphasizes the deleterious combination of metS- and diastolic dysfunction-induced PH-HFpEF through activation of the IL-6/STAT3 pathway as a key mechanism of PV remodeling.

### Diabetes-Related Metabolic Cardiomyopathy

Metabolic cardiomyopathy has been demonstrated to result from metS diseases, such as obesity and type 2 diabetes. This remodeling predisposes structural and functional changes that lead to HFpEF. A key player in this process are sirtuins, which are molecules that maintain metabolic homeostasis by regulating fatty acid metabolism and mitochondrial function, both maladaptations that have been linked with HFpEF (70). In fact, the beneficial effects of sodium glucose transporter-2 inhibitors in HFpEF have been suggested to be due to restoration of sirtuin-1 (SIRT1) activity (71). Costantino et al. (70) demonstrated that dietary supplementation with recombinant SIRT1 preserved cardiac function and restored cardiac lipidome remodeling in murine models of metabolic cardiomyopathy, reducing incidence of HFpEF.

### Lipotoxicity

Obesity has a central role in the development of HFpEF. A causal link between adiposity and mediators of inflammation has been recognized through a mechanism known as “lipotoxicity” (72). Lipotoxicity suggests that lipid accumulation from nonadipose organs results in oxidative stress, mitochondrial dysfunction, and apoptosis, leading to skeletal muscle insulin resistance, pancreatic β-cell dysfunction, and nonalcoholic fatty liver disease in metS (72). Ranchoux et al. (64) demonstrated a direct correlation between ectopic adipose tissue accumulation and PH severity in animals with metS and diastolic dysfunction. In addition, the association between metS adiposity and PH-HFpEF was established via the development of a mouse model on high-fat diet (65). This mouse model demonstrates several clinical features of PH-HFpEF, including elevated RV systolic pressure, LV end diastolic pressure, preserved LVEF, and biventricular hypertrophy (65). Specifically, leptin is a molecule made from fat cells directly related to adiposity that is closely associated with metS and diastolic function (73). Prior studies have activated the leptin/leptin receptor axis, which contributes to PASMC proliferation and abnormal macrophage activation (74).

### Regulatory T Cells/T Helper 17 Cell Balance

It has been well established that cpc-PH attributed to HFpEF mimics the etiology of WHO Group I PAH (17, 75); in fact, research has also demonstrated how ET-1 receptor antagonization resulted in a significant reduction of PVR and improved exercise capacity in both cpc-PH attributed to HFpEF and PAH (42). Therefore, examining the inflammatory mechanisms in PAH may elucidate the pathogenesis of cpc-PH.

Equilibrium and homeostasis of the immune response in WHO Group I PAH has been shown to primarily involve T helper 17 (Th17), regulatory T (Treg), and cytotoxic T (Tc) cells (76). Although Th and Tc cells produce a proinflammatory response, Treg cells produce an anti-inflammatory response. Human and animal models have shown that the imbalance of Treg:Th17 ratio correlates with PAH disease severity (76). Treg cells and their anti-inflammatory byproducts regulate growth and hypertrophy of the vasculature and smooth muscle cells under inflammatory conditions via suppressing the Akt/ERK pathway, which is the pathway responsible for triggering cell growth, survival, and motility (77). Through this pathway, Treg cells have suppressive effects on collagen and fibroblasts and control ventricular remodeling, preventing development of RV hypertrophy (78). In addition, Treg dysfunction has been directly shown to increase PAH progression via decreasing inflammatory effects (79). Treg dysfunction could also explain the RV maladaptive remodeling in cpc-PH. Treg:Th17 imbalance has been demonstrated to exist in patients with chronic HFpEF in the study by Li et al. (80), but these results were also seen in patients with HFrEF.

### Macrophage Proliferation

Agrawal et al. (81) demonstrated that as early as 2 wk of exposure to *N*^G^-nitro-l-arginine methyl ester/high-fat diet, mice develop PH-HFpEF features, including preserved CO, diastolic dysfunction, weight gain, increased PVR, RV systolic pressure, RV mass, and histological PV remodeling. The same model exposed to clodronate (to deplete monocytes and macrophages) demonstrated less weight gain, near normal RV systolic pressure, normal RV mass, and decreased PV remodeling (81). Studying the cytokine profile further, the study inhibited IL-1B to yield the same changes, emphasizing the unique and specific role of myeloid produced IL-1B in small vessel muscularization (81). These results suggest the role of circulating myeloid cells that migrate to the lungs and contribute to PH and RV dysfunction in PH-HFpEF. In addition, galectin-3, a lectin expressed by activated cardiac macrophages, has also been shown to be associated with adverse LV remodeling, cardiomyocyte hypertrophy, and myocardial fibrosis (82). In fact, galectin-3 has been shown to be an independent prognostic marker in humans with PH-HFpEF (83).

### Metabolic Interventions

Many of the current treatments that exist for PH-HFpEF rely on treating comorbid metS. As previously discussed, anti-IL-6-antibodies, metformin, and nitrite provide promising results in animal models of PH-HFpEF (63, 65). The role of metformin in PV remodeling is interesting. Metformin treatment has been shown to improve PH-LHD, either by inhibiting leptin secretion or by inhibiting aromatase/estrogen synthesis (64, 84). Metformin failed to reverse PV remodeling in mice with SuHx (combined exposure of hypoxia and vascular endothelial growth factor receptor antagonist)-induced PAH, although treatment prevented development of PAH in hypoxia models (84). Many patients with PAH present with signs of insulin resistance and glucose intolerance sans obesity and type 2 diabetes that are more prevalently seen in PH-HFpEF; therefore, the divergent responses to early animal clinical trials suggest preferentially beneficial treatment of PH-HFpEF, with limited treatment efficacy for PAH (84). Antidiabetic agents such as sodium glucose transporter-2 inhibitors and glucagon-like peptide-1 receptor agonists are currently included in the guideline-directed medical therapy for HFpEF (18). Recent trials with Semaglutide exhibited reduced symptoms, improved exercise function, and induced greater weight loss in patients with HFpEF and obesity (85). Although promising, their role in the treatment of PH-HFpEF entails further study.

## NOVEL MECHANISMS OF PH-HFpEF: POLYUNSATURATED FATTY ACIDS IN REGULATING CARDIOVASCULAR INFLAMMATION AND REMODELING

Polyunsaturated fatty acids (PUFA), arachidonic acids (AA), and their metabolites play an important role in regulating complex cardiovascular functions under physiological and pathological conditions. O’Sullivan et al. (86) recently demonstrated how in the nonfasting state, HFpEF hearts transition to complex lipid generation correlating with increasing PA pressures. Furthermore, Jovanovic et al. (87) examined the human plasma lipidome in patients with HFpEF to discover significant correlation between smoking status and individual long-chain fatty acid lipid species (despite no difference between disease and control groups), and significant differences between distinct lipid species and echocardiographic parameters in HFpEF. Although the group could not conclude significant associations between lipidomic parameters and HFpEF, limited by their sample size, the study suggests a complex, nonlinear relationship between lipid metabolism and disease progression that entails further study (87). Indeed, our group has also noted several potential mechanistic links between these inflammatory mediators and PH-HFpEF, including discovering multiple PUFA metabolites significantly predictive of ipc-PH, cpc-PH, and no PH, and metabolites that predicted all cause hospitalization and mortality (88, 89). In this section, we review novel aspects of PUFA lipid mediators and potential links to the adverse PV remodeling and RV dysfunction seen in cpc-PH attributable to HFpEF, as outlined in Table 3.

**Table 3.** Suggested role of oxylipins in PH-HFpEF

PH Hallmarks Oxidized Lipids	Effect	References
Vascular remodeling		
COX-derived PGE_2_	Mediates neointimal hyperplasia via cyclic stretch upregulation of COX-2	(38)
CYP-derived EET	Protects against myocardial structural and metabolic remodeling	(90)
Apoptosis		
LOX-derived 15-HETE	Activates SIRT1 to protect against PASMC apoptosis	(70, 91)
LOX-derived maresins	Improves RV systolic pressure by inhibiting the AKT/Erk pathway and improving Treg/Th17 imbalance	(92)
Vasoconstriction		
COX-derived PGI_2_	Reduces PASP by promoting pulmonary artery relaxation and improves RV hypertrophy and PV intimal wall thickening	(93)
COX-derived TXA_2_	Antagonizes PGI_2_ actions and acts synergistically with serotonin with mitogenic effect on PASMCs	(94)
Inflammation		
COX-derived TXA_2_	Stimulates platelet aggregation	
LOX-derived LXA_4_	Improves RV systolic pressure by decreasing IL-10 and ROS generation in vascular endothelial cells to improve RV systolic pressure	(95, 96)
CYP-derived EET	Decreases TGF-β profibrotic activity	(97)
LOX-derived D-series resolvins	Suppresses neutrophil migration and cytokine production by activating innate immunity and promoting phagocytotic activity in macrophages	(98)

COX, cyclooxygenase; EET, epoxyeicosatrienoic acid; 15-HETE, 15**-**hydroxyeicosatetraenoic acid; IL-10, interleukin-10; ROS, reactive oxygen species; LOX, lipoxygenase; LXA_4_, lipoxin A_4_; PASMCs, pulmonary arterial smooth muscle cells; PASP, pulmonary artery systolic pressure; PGE_2_, prostaglandin E_2_; PGI_2_, prostacyclin; PH-HFpEF, pulmonary hypertension in the setting of heart failure with preserved ejection fraction; PV, pulmonary vascular; ROS, reactive oxygen species; RV, right ventricular; SIRT1, sirtuin1; TGF-β, transforming growth factor-β; Treg/Th17, regulatory T cells/T helper 17 cells; TXA_2_, thromboxane A_2_.

**Figure 4.**
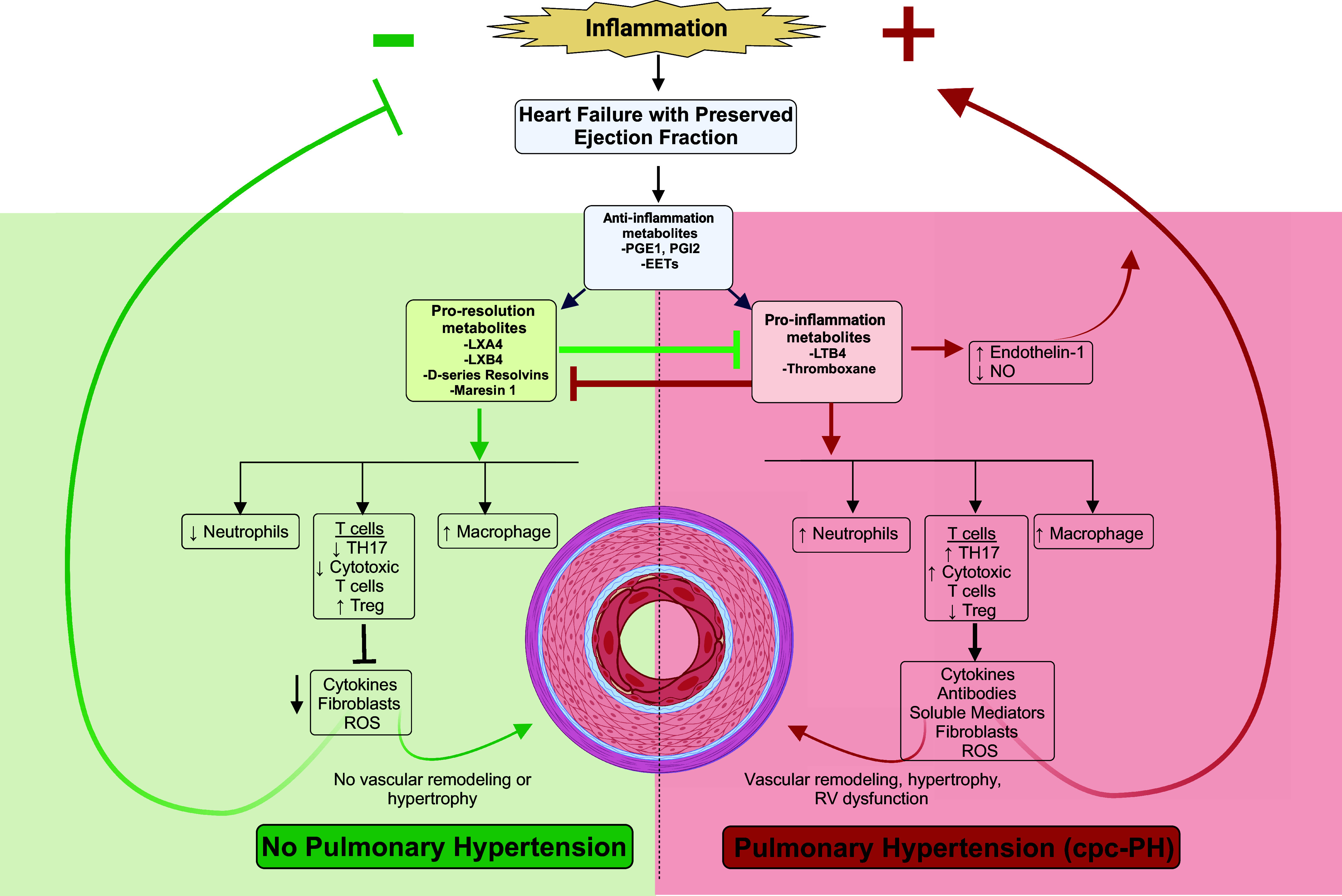
Role of inflammatory lipid homeostasis in pulmonary hypertension (PH)-heart failure (HF) with preserved ejection fraction (PH-HFpEF). Diagram shows the balance of inflammatory lipid mediator homeostasis that is needed to prevent progression to PH-HFpEF. The comorbidities present with HFpEF establish a baseline state of chronic low-grade inflammation, defined by IL-6 activation, macrophage and neutrophil proliferation, and imbalance of regulatory T cells (Treg)-to-T helper cells (Treg:Th17) ratio. This chronic inflammatory state can be characterized and enhanced by the imbalance of anti-inflammatory lipid metabolites with proresolutory metabolites and proinflammatory metabolites. In a state where the balance is tipped toward proinflammatory lipid metabolism, we predict that this can further increase proliferation of immune cells and cytokines, antibodies, soluble mediators, fibroblasts, and reactive oxygen species (ROS), predisposing to a complex state of the deleterious pre- and postcapillary PH (cpc-PH) in patients with HFpEF. Conversely, we predict that the predominance of proresolutory metabolites can protect against this state, as seen in patients with compensated HFpEF who do not develop cpc-PH or develop isolated postcapillary PH (ipc-PH). PGE_1_, prostaglandin E_1_; PGI_2_, prostacyclin; LXA_4_, lipoxin A_4_; LXB_4_, lipoxin B_4_; LTB_4_, leukotriene B_4_; NO, nitric oxide; ROS, reactive oxygen species; RV, right ventricular. Figure was created with a licensed version of BioRender.com.

*N*-3 (omega-3) PUFAs have been found to be effective in reducing ischemic heart disease, atrial and ventricular arrhythmia, hypertension, peripheral artery disease, and HF; furthermore, a study carried out by Hassan and Hanachi group showed that a *n*-3 PUFA rich diet can decrease likelihood of developing metS (99). Almost all lipid mediators derived from *n*-3 PUFA are proresolutory mediators of inflammation. Conversely, *n*-6 (omega-6) PUFAs, such as AA, are precursors to primarily proinflammatory mediators, with studies demonstrating higher intakes of *n*-6 PUFA associated with increased IL-6 and CRP (100). Increase in *n*-6:*n*-3 ratio is seen in chronic inflammatory diseases, such as cardiovascular disease, obesity, inflammatory bowel disease, and nonalcoholic fatty liver disease. AA and its metabolites are the most studied and emphasized in relevance to cardiovascular biology. Generally, AA metabolism by cyclooxygenases (COX), lipoxygenases (LOX), and cytochrome-P450 (CYP) enzymes leads to mediators of proinflammation. These enzymes can then generate a vast spectrum of oxidized lipid mediators, generally referred to as “oxylipins” to mediate inflammatory processes and disease (Fig. 4). Although AA-derived oxylipins are primarily proinflammatory, they can also be anti-inflammatory or proresolutory. Almost all proresolution lipid mediators are derived from the LOX pathway, with leukotrienes (LTs; e.g., LTB_4_) as an exception. COX, LOX, and CYP enzymes also metabolize *n*-3 PUFAs to generate proresolutory and anti-inflammatory oxylipins such as eicosapentanoic acid (EPA) and docosahexaenoic acid (DHA).

### Cyclooxygenase

Differentiation of fibroblasts into proinflammatory myofibroblasts is a critical mechanism in arterial fibrosis that characterizes the irreversible remodeling of cpc-PH. Cyclic stretch has been shown to increase the expression of fibroblast growth factors in PASMCs, and upregulate cyclooxygenase (COX)-2 (38). COX-2 derived prostaglandin-E_2_ (PGE_2_) has been demonstrated to play a role in neointimal hyperplasia, as targeted deletion of PGE_1_ receptors in vascular smooth muscle cells directly impacts vascular remodeling in mice (93). PGI_2_ has been shown to reduce PASP by promoting PA relaxation, and current treatment for PAH remains PGI_2_ analogs, such as iloprost, treprostinil, and epoprostenol. In fact, even in patients with HFpEF, preliminary results show that inhaled iloprost favorably improves myocardial performance during exercise by improving RV systolic function (101). In patients with PH-HFpEF, treprostinil has also been suggested as an early treatment for mild metS-associated PH-HFpEF, with similar effects as metformin (102). Past studies have shown how treatment with PGE_1_ via epoprostenol reduces the maladaptive vascular remodeling in the lungs and heart, and improves RV hypertrophy and intimal wall thickening via potent alveolar vasodilation in rat models of PH (103). COX-derived PGE_1_ and PGE_2_ have also been used to treat postoperative PH (e.g., mitral valve replacement or open-heart surgery) and improve ventricular function and survival in PH secondary to HF (103).

Proinflammatory COX-derived thromboxane A_2_ (TXA_2_) is a potent stimulus for platelet aggregation, directly antagonized by the actions of PGI_2_. The imbalance between the release of TXA_2_, a vasoconstrictor, and PGI_2_, a vasodilator, may contribute to the development of cpc-PH. Although data regarding the ability of TXA_2_ to stimulate PASMC proliferation are inconsistent, it has been shown that the presence of serotonin is synergistic with TXA_2_ production, with a possible mitogenic effect on PASMCs (94). These changes in blood vessel walls can lead to reduced diameter of pulmonary arteries, and over time, right HF.

### Lipoxygenase

Lipoxygenase (LOX)-derived 15-hydroxyeicosatetraenoic acids (15-HETE) plays a significant role in hypoxic PAH via vascular remodeling and proliferation of PASMCs through the protein kinase C signal transduction pathway (104). Although 15-HETE has not been directly studied in association with PH-LHD, studies have demonstrated that 15-HETE promotes SIRT1 levels in PASMCs (91). SIRT1 has been previously demonstrated as a key player in HFpEF pathogenesis (70). SIRT1 also mediates 15-HETE-induced PASMC proliferation during hypoxia and normoxia, protecting PASMC apoptosis and remodeling (91). Further study regarding 15-HETE and vascular remodeling in PH-HFpEF is necessary, as this mechanism can constitute a new therapeutic foundation.

Lipoxin A_4_ (LXA_4_) and LXB_4_ attenuate the inflammatory response by leading to downstream production of IL-10, a regulatory cytokine, which can further reduce adhesion and aggregation of neutrophils (95). LXA_4_ demonstrates atheroprotective effects via impairing production of proinflammatory cytokines, inhibiting neutrophil chemotaxis, and activating the proresolving functions of macrophages (105). In addition, studies have demonstrated that LXA_4_ suppresses NADPH-oxidase mediated ROS generation in vascular endothelial cells (105). The cardioprotective nature of statins can also be partly attributable to LXA_4_; cholesterol reducing medications such as HMG-CoA reductase inhibitors atorvastatin and simvastatin have been shown to increase the myocardial content of LXA_4_ (96). LOX metabolism of *n*-3 DHA can produce proresolutory maresins and D-series resolvins. Resolvins attenuate ROS generation, inhibit cytokine release, stimulate macrophages to phagocytose neutrophils, and disrupt TX-mediated platelet aggregation (98). Our group has specifically identified maresin-1 as a key lipid metabolite predictive of cpc-PH in HFpEF (88). Maresin-1 suppresses neutrophil migration and cytokine production by activating Tc, Th1, and Treg cells, and promoting phagocytotic activity in macrophages. Maresin-1 has also been shown to improve Treg:Th17 imbalance in rheumatoid arthritis, an imbalance that has been suggested to propagate HFpEF (80, 92). Maresin-1 intervention has been shown to reverse PAH in mice by decreasing RV systolic pressure and RV dysfunction through inhibiting the AKT/Erk pathway, the pathway responsible for PV remodeling (106). This demonstrates that maresin-1 may have a potent therapeutic effect in the cpc-PH component of HFpEF.

### Cytochrome-P450

Produced primarily in the endothelium and myocardium, CYP-generated epoxyeicosatrienoic acids (EETs) function as anti-inflammatory mediators by reducing systemic hypertension and promoting angiogenesis. EETs function as microcirculatory vasodilators in arteries, and low levels are associated with endothelial dysfunction and decreased NO bioavailability (90). Animal models of cardiac hypertrophy and HFpEF display low-cardiomyocyte EET level, suggesting the protective effect of EETs against myocardial structural and metabolic remodeling (90). Finally, cardiac fibrosis is a primary pathological change in HFpEF leading to diastolic dysfunction; indeed, EETs relieve the differentiation of cardiac fibroblasts into myofibroblasts via inhibition of transforming growth factor-β (TGF-β), which has been shown as the key transformation in cpc-PH (38, 97, 107). Recent studies also demonstrated how EETs reduce PH and lung ischemia-reperfusion injury in rats with PH-LHD (108). EETs pose as promising candidates for treating PH-HFpEF.

### Quantifying PUFA Metabolites for Prognostic Scoring of PH-HFpEF

Given the immense biological diversity of PUFA-derived oxygenated metabolites that carry potential for significant clinical utility, there is a need for a sensitive, selective, reliable, and accurate assay system to assess lipid species quantitatively and qualitatively. Lipid chromatography mass spectroscopy (LC-MS) is the method of choice to quantify the hundreds of metabolites and create a risk stratification score based on the contribution of each molecule to the severity of PH-HFpEF progression. LC-MS is a reliable analytical platform for mediator lipidomic assays with detection limits even below their physiological activities (in the subpicogram range). Given that certain PUFA metabolites partake in anti-inflammatory, proinflammatory, and proresolutory processes, it is possible to stratify a risk and severity score from populations with PH-HFpEF and potentially detect early prognostic and diagnostic biomarkers for early development of PH without the need for invasive RHC.

## CONCLUSION

Development of PH in HFpEF is a significant prognostic indicator of increased short- and long-term mortality risk. The prevalence of HFpEF increases with age and is strongly associated with female gender and chronic metS encompassing obesity, type 2 diabetes mellitus, hypertension, pulmonary disease, and liver disease; therefore, it is likely that patients with HFpEF sustain a chronic, low-grade inflammatory state. We suggest that the inflammatory state activates neurohormonal mediators, adaptive immunity, and PUFA-generated lipid enzymatic metabolism, which leads to PV remodeling, pulmonary vasoconstriction, and RV diastolic dysfunction. These changes finally contribute to cpc-PH progression and subsequent clinical worsening. Elucidating the critical knowledge gaps in pathophysiology is essential to discovering new pharmacological strategies targeting noncardiac biomarkers to improve PH-HFpEF outcomes. Quantitative and qualitative lipidomics analysis opens a new frontier in the pathophysiology of HFpEF and holds significant promise for diagnostic and therapeutic advancements in PH-HFpEF.

## GRANTS

This work was supported by the Adela and Alfred Mundt Foundation for Heart Failure Research (to S.J.K.); National Heart, Lung, and Blood Institute Grant HL-137004 (to D.J.K. and S.T.H.); the David and Helen Boone Foundation Research Fund (to D.J.K.); and the University of Toledo Women and Philanthropy Genetic Analysis Instrumentation Center (to D.J.K. and S.T.H.).

## DISCLOSURES

No conflicts of interest, financial or otherwise, are declared by the authors.

## AUTHOR CONTRIBUTIONS

V.A., D.J.K., and S.J.K. conceived and designed research; V.A. prepared figures; V.A., R.V., R.G., D.J.K., and S.J.K. drafted manuscript; V.A., R.V., P.D., S.T.H., R.G., K.R.M., D.J.K., and S.J.K. edited and revised manuscript; V.A., R.V., P.D., S.T.H., R.G., K.R.M., D.J.K., and S.J.K. approved final version of manuscript.
